# Optimal screening and donor management in a public stool bank

**DOI:** 10.1186/s40168-015-0140-3

**Published:** 2015-12-17

**Authors:** Abbas Kazerouni, James Burgess, Laura J. Burns, Lawrence M. Wein

**Affiliations:** Electrical Engineering Department, Stanford University, Stanford, CA, 94305 USA; OpenBiome, 196 Boston Avenue, Medford, MA, 02155 USA; Graduate School of Business, Stanford University, Stanford, CA, 94305 USA

**Keywords:** Fecal microbiota transplantation, Clostridium difficile, Mathematical modeling

## Abstract

**Background:**

Fecal microbiota transplantation is an effective treatment for recurrent Clostridium difficile infection and is being investigated as a treatment for other microbiota-associated diseases. To facilitate these activities, an international public stool bank has been created, which screens donors and processes stools in a standardized manner. The goal of this research is to use mathematical modeling and analysis to optimize screening and donor management at the stool bank.

**Results:**

Compared to the current policy of screening active donors every 60 days before releasing their quarantined stools for sale, costs can be reduced by 10.3 % by increasing the screening frequency to every 36 days. In addition, the stool production rate varies widely across donors, and using donor-specific screening, where higher producers are screened more frequently, also reduces costs, as does introducing an interim (i.e., between consecutive regular tests) stool test for just rotavirus and C. difficile. We also derive a donor release (i.e., into the system) policy that allows the supply to approximately match an exponentially increasing deterministic demand.

**Conclusions:**

More frequent screening, interim screening for rotavirus and C. difficile, and donor-specific screening, where higher stool producers are screened more frequently, are all cost-reducing measures. If screening costs decrease in the future (e.g., as a result of bringing screening in house), a bottleneck for implementing some of these recommendations may be the reluctance of donors to undergo serum screening more frequently than monthly.

**Electronic supplementary material:**

The online version of this article (doi:10.1186/s40168-015-0140-3) contains supplementary material, which is available to authorized users.

## Background

Recurrent and refractory Clostridium difficile infection (CDI) is a leading cause of hospital-acquired infection [1], leading to 30,000 deaths [2] and $4.8 B in hospital costs [3] annually in the USA. Although frontline treatment with metronidazole or vancomycin have suffered from increasing failure rates in recent years [4], fecal microbiota transplantation (FMT), i.e., stool transplanted from a healthy donor that reconstitutes the normal microbiota community in the gut, has emerged as an effective treatment, with a cure rate of 90 % in recurrent cases [5].

A nonprofit organization, OpenBiome, has built an international public stool bank, where donor stool is screened and processed in a standardized manner with the goal of facilitating safe FMTs for the treatment of recurrent CDI and as a platform for investigating other microbiome-associated diseases. Their stool has been distributed to over 415 clinical institutions in 49 US states and six countries (www.openbiome.org/impact).

There are several logistical challenges associated with managing a stool bank. First and foremost, screening of potential donors and of stool and serum specimens is vital for preventing the transfer of infectious diseases and to mitigate the theoretical risk of making recipients more susceptible to chronic conditions such as obesity or autoimmune disorders [6]. In addition, donors need to be dynamically released into the system so that the supply of stool approximately matches a rapidly growing demand. We use OpenBiome data to build a mathematical model that tracks the process flow of stools from donation to sale. This model is embedded into an optimization problem that releases new donors and chooses the donor-specific frequency of stool and blood screening to minimize donating, processing and screening costs subject to meeting an exponentially increasing demand.

## Methods

### The mathematical model

**Fig. 1:**
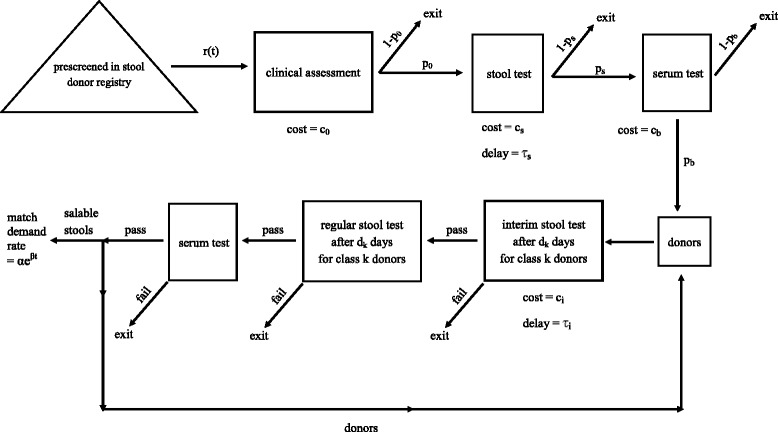
The process flow

The process flow is diagrammed in Fig. 1, and the model’s parameters and their values are given in Table 1. Potential participants join a stool donor registry, and 55 % of these people pass the prescreen that rules out common exclusion factors such as age, body mass index, antibiotic use, travel history, birth country, or donation logistics [7]. Because donor registration and prescreening are fully automated, have no variable cost, and are not a bottleneck in the process, we assume that there are an ample number of prescreened people in this registry. The first of three control variables in our model is denoted by *r*(*t*), which is the rate at which prescreened potential participants are taken from the stool donor registry and released into the system at time *t* (although we use a continuous time model, the data are given in days and so we sometimes refer to day *t*). Upon being released into the system, a prescreened potential participant completes an on-site 109-question clinical assessment conducted by a gastroenterologist and a qualified research nurse [7] to rule out risk factors for transmissible diseases and potential microbiome-related conditions, such as high-risk sexual behavior, psychiatric conditions, and autoimmune diseases [7]. The proportion of prescreened potential participants who pass the clinical assessment is denoted by *p*_0_, and the cost of the clinical assessment is *c*_0_ per person. Potential participants passing the clinical assessment then undergo a stool test, and those passing the stool test undergo a serum test, where these two combined tests consist of 27 stool-based and serological assays for detection of communicable infectious agents. The proportion of people passing the stool test and serum test are *p*_*s*_ and *p*_*b*_, and the per person costs of the two tests are *c*_*s*_ and *c*_*b*_, respectively. In addition, the serum test generates same-day results (and so we ignore any delay), but the stool test results incur a delay of *τ*_*s*_ days.

**Table 1:** The model’s parameters, along with their descriptions and values

Parameter	Description	Value
*p*_0_	Proportion who pass clinical assessment	0.351
*p*_*s*_	Proportion who pass first stool test	0.444
*p*_*b*_	Proportion who pass first serum test	1.0
*c*_0_	Cost of clinical assessment	$50
*c*_*s*_	Cost of stool test	$600
*c*_*b*_	Cost of serum test	$235
*c*_*i*_	Cost of interim test	$120
*c*_*d*_	Cost of paying donors	$18/day
*c*_*p*_	Cost of processing donated stool	$0.15/g
*τ*_*s*_	Time delay of stool test	12 days
*τ*_*i*_	Time delay of interim test	5 days
*f*_1_,…,*f*_*n*_	PMF of donor stool production rate	Section 1.1 in Additional file 1
*η*	Exponential failure rate for Rotavirus and C. difficule	0.0066/day
*γ*	Exponential failure rate for the other 25 agents	0.0040/day
*T*	Time horizon of optimization problem	365 days
*α*	Initial demand rate	240 g/day
*β*	Exponential demand parameter	0.0029/day

Participants passing the stool and serum screens immediately begin donating stool, and donated samples (greater than 55 g and scoring between 3 and 5 on the Bristol scale, page 240 in [8]) are collected within 1 h, suspended in a sterile saline and glycerol solution, homogenized, filtered to 330 *μm*, and stored at −80 °C [9]. Donors are paid *c*_*d*_ per day until they exit the system, and it costs *c*_*p*_ per gram to process donated stool. Each donor has a random stool production rate in grams per day, which has a probability mass function (PMF) *f*_*k*_, which is the probability that a donor’s stool production rate equals *s*_*k*_, for *k*=1,…,*n*. For simplicity, we assume that each donor produces stool at a continuous rate, even though not every donor donates stool every day at OpenBiome. Donors with stool production rate *s*_*k*_ are referred to as donors of class *k* for *k*=1,…,*n*. Because each donor’s stool production rate can be estimated rather quickly relative to the time between screens, we assume in our model that a donor’s class is observable. Our second set of decision variables is the inter-testing time *D*_*k*_ (in days), which is allowed to vary by class, that is, after every *D*_*k*_ days of donating, a donor of class *k* undergoes another identical round of stool and serum tests; these tests are referred to as regular tests, so as to distinguish them from the interim tests introduced later. To prevent the transmission of infectious diseases, the donated stool is quarantined until the donor passes a new round of testing. Hence, if a donor of class *k* passes a new round of tests on day *t*, then the donor’s donations over the previous *D*_*k*_ days are released from quarantine and offered for sale, and the donor continues to donate. If a donor fails the new round of tests, the donor’s donations over the previous *D*_*k*_ days are discarded, and the donor is ejected from the system (i.e., the donor is no longer allowed to donate). In addition, the two tests are given sequentially, and the serum test is not performed if the donor fails the stool screen.

Many of the test failures are due to two of the 27 agents, rotavirus and Clostridium difficile, and we assume that the time until failure (i.e., the time from passing the initial serum screen to developing an infection that would be detected by the subsequent stool or serum tests) is an exponential random variable with rate *η*+*γ*, where *η* is the failure rate associated with the two specific agents, and *γ* is the failure rate associated with the other 25 agents. Our final set of decision variables allows for the possibility of performing an interim stool test for only these two agents, which costs *c*_*i*_ per person and has a testing delay of *τ*_*i*_ days; although serum tests are obviously transparent to the donor, the OpenBiome patient consent policy allows additional stool testing without notification, and so interim stool testing would likely be performed without transparency. For a class *k* donor, the interim test occurs *d*_*k*_ days after a donor starts donating and *d*_*k*_ days after each successful regular test, where *d*_*k*_<*D*_*k*_; e.g., if a class *k* donor started donating at time *t*, and *D*_*k*_=60 days and *d*_*k*_=22 days, then interim tests would occur at time *t* + 22 and—if the donor passed the regular tests at time *t* + 60—at time *t*+82, and so on. Even if a donor passes the interim tests, the donor’s samples remain in quarantine until the donor passes a regular test that screens for all 27 agents. If a donor fails an interim test, then the quarantined samples are discarded and the donor is ejected from the system.

Because FMT demand was rapidly increasing throughout the time period of our study, we assume that the demand rate at time *t* is *αe*^*βt*^ g/day for *t*∈ [ 0,*T*], where *T* is the time horizon of our model. We assume that demand is deterministic, and view *αe*^*βt*^ as a target trajectory over *t*∈ [ 0,*T*].

The optimization problem is to choose the donor release rate *r*(*t*), the time between consecutive regular tests *D*_*k*_,*k*=1,…,*n*, and the time between a regular test and an interim test *d*_*k*_,*k*=1,…,*n* to minimize all costs over *t*∈ [ 0,*T*] associated with screening, donating and processing. Because demand is deterministic, we require that demand be exactly satisfied for all *t*∈ [ 0,*T*] (i.e., the rate at which salable stool is released from quarantine must equal the demand rate *αe*^*βt*^).

### Parameter estimation

**Fig. 2:**
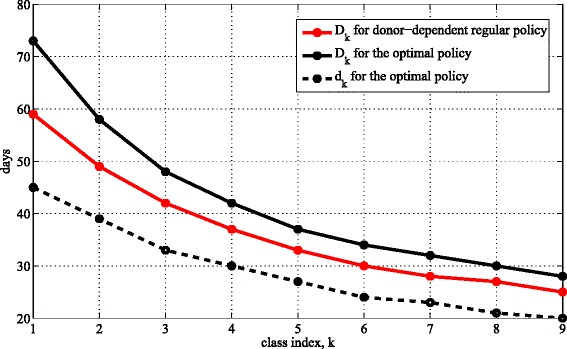
The optimal donor-dependent screening variables. The optimal inter-testing time for regular tests (*D*_*k*_) and the optimal time between a regular test and an interim test (*d*_*k*_) as a function of a donor’s class, where classes *k*=1,…,9 have stool production rates in the intervals (4.7−25.3,25.3−45.9,45.9−66.5,66.5−87.1,87.1−107.7,107.7−128.3,128.3−148.9,148.9−169.7,169.7−190.3) g/day

In [7], 27 of 77 people passed the clinical assessment, 12 of 27 passed the initial stool test, and all 12 passed the initial serum test, giving *p*_0_=27/77=0.351, *p*_*s*_=12/27=0.444, and *p*_*b*_=1.0. The clinical assessment takes 1.5 h, for an estimated cost of $50. The stool and serum tests are currently performed by outside laboratories, and the costs listed in Table 1 include a $60 payment to donors for getting a serum test. Stool tests take *τ*_*s*_=12 days and interim stool tests take *τ*_*i*_=5 days. Processing a stool takes up to 1 h for a cost of $30, and donors are paid $40 per stool. The mean stool size is 194.0 g, and the mean stool production rate is 87.2 g/day, implying that donors donate an average of 87.2/194=0.45 stools per day. Hence, the processing cost is *c*_*p*_=30/194=*$*0.15/ g and the donation cost is 0.45(*$*40)=$18/day. We fit a PMF with *n*=9 classes (with *s*_*k*_ values ranging from 15 to 180 g/day for *k*=1,…,9) using stool production data from 30 donors in Section 1.1 and Fig. 1 in Additional file 1. We also find that the number of weekly visits per donor does not vary over his or her lifetime as a donor (Section 1.1 and Fig. 2 in Additional file 1); in particular, there is no evidence of donor fatigue (i.e., the visit rate waning over time). Maximum likelihood estimation is used to fit test results from 13 donors in Section 1.2 of Additional file 1 to estimate *η* and *γ* in Table 1.

We consider the stool bank operations over *T*=365 days, where the growth rate of demand is 10 % per month. Setting *e*^365*β*^ equal to 1.1^11^ gives the exponential growth parameter *β*=0.0029/day. The target demand rate at the end of the year is set equal to 10 % of potential demand for recurrent CDI. Assuming 500,000 new CDI cases per year [2] and a 20 % recurrence probability [10], the target demand is 10,000 cases/year. We assume 25 g of stool per treatment via colonoscopy (250 mL per treatment at a concentration of 10 mL/g). Hence, the demand rate at the end of the year is 10,000 cases/year × 25 g/case × 1 year/365 days =685 g/day. Setting *αe*^0.0029(365)^=685 gives *α*=240 g/day.

## Results

In Section 2.1 of Additional file 1, we show that the optimization problem described above can be explicitly written as a convex program (and hence easily computed) for the decision variables (*D*_1_,…,*D*_*n*_,*d*_1_,…,*d*_*n*_) in the case of stationary demand (i.e., *β*=0). In Section 2.2 of Additional file 1, we further show that the optimal solution (*D*_1_,…,*D*_*n*_,*d*_1_,…,*d*_*n*_) to the problem when *β*>0 is the same as in the case with *β*=0. To assess the improvements from optimal screening relative to the status quo, and to identify the importance of interim testing and the importance of allowing testing frequency to depend on donor-specific stool production rates, we consider the five policies in Table 2, which include the status quo policy used by OpenBiome (*D*=60 days and no interim testing [9]) and the optimal policy to the optimization problem described above. The other three policies are restricted forms of the optimal policy, where we disallow the use of either interim testing or testing frequencies that depend on stool production rates. The optimal policy achieves a 14.5 % reduction in cost relative to the status quo policy (Table 2). However, more than two thirds of this reduction can be achieved by simply reducing the inter-testing time for regular tests from 60 to 36 days. Interim testing is of only modest value when testing times are not donor-dependent. Interim testing causes only a minor increase (from 36 to 41 days) in the inter-testing time of regular tests and occurs ≈70 *%* of the way through the donation cycle (i.e., *d*=29 days, *D*=41 days).

**Table 2:** The main results. The five policies, along with their absolute and relative costs

Screening policy	Inter-testing time	Time between interim	Annual cost	Cost reduction
	for regular tests	and regular tests		vs. status quo (%)
Status quo	*D*=60 days	No interim testing	$209,148	–
Fixed regular	*D*=36 days	No interim testing	$193,137	10.3
Fixed	*D*=41 days	*d*=29 days	$190,645	11.9
Donor-dependent regular	*D*_*k*_ in Fig. 3	No interim testing	$189,679	12.5
Optimal	*D*_*k*_ in Fig. 3	*d*_*k*_ in Fig. 3	$186,548	14.5

When inter-testing times are allowed to vary by donor, it is optimal to screen high-producing donors more frequently (Fig. 2), with the highest-producing donor class being screened more than twice as frequently as the lowest-producing donor class. As in the donor-independent cases in Table 1, interim testing occurs most of the way through the testing cycle and increases the regular inter-testing times by only a modest amount.

**Fig. 3:**
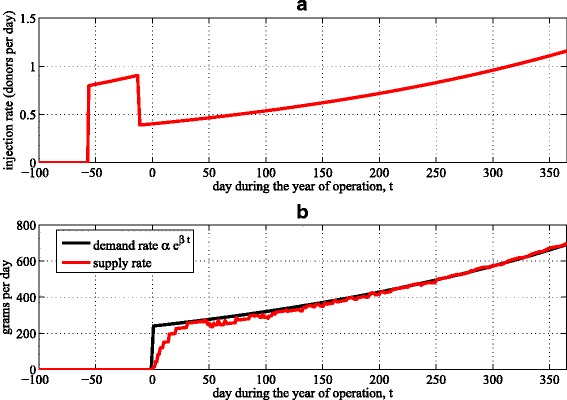
Assessing the donor release policy over the year, *t*∈[0,365]. **a** The approximate donor release policy derived in Eq. (40) in Additional file 1. **b** The supply rate of salable stool resulting from the release policy in (**a**), and the demand rate, *α* *e* ^*βt*^

From an implementation standpoint, the optimal policy in Fig. 2 is pushing up against one impediment: it calls for serum screening of 30 % of donors—the higher-producing donors—at least monthly, and OpenBiome management believes that—because of the required blood draw—donors will perceive serum screening more frequently than monthly as too onerous. Consequently, to investigate whether more frequent interim (stool) screening may be beneficial, we recompute the optimal screening policy with the additional constraint *D*_*k*_≥30 days and under two scenarios: class 9 is required to have a second interim test and classes 6–9 are required to have a second interim test. Relative to the optimal screening policy in Table 2, the cost increases by 0.2 and 1.3 %, respectively, under these two scenarios (the screening policies in these two scenarios appear in Fig. 3 in Additional file 1). Hence, more frequent interim tests do not reduce costs.

We also assess the following two-parameter policy that is easier to implement than the optimal policy because it does not require categorizing donors by their stool production rates: for *k*=1,…,9, set *d*_*k*_=*x*/*s*_*k*_ and *D*_*k*_=*y*/*s*_*k*_, that is, this policy performs an interim test on each donor after *x* grams of stool have been produced and performs a regular test on each donor after *y* grams of stool have been produced. The optimal solution is *x*=1899 g and *y*=3201 g (Fig. 4 in Additional file 1), which increases the cost by 4.2 % relative to the optimal policy in Table 2. Hence, this simplification of the optimal policy comes at a significant cost.

The results for the donor release rate are derived in Section 2.3 in Additional file 1. In the stationary demand case (i.e., *β*=0), the donor release rate that generates salable stool at exactly the demand rate is given in Eq. (12) in Additional file 1, and the release rate *r* and the demand rate *α* are related via *r*=*α*/958, where 958 represents the expected total number of salable grams of stool produced by a released donor (i.e., someone who is prescreened in the donor registry). In the nonstationary demand case, the donor release rate *r*(*t*) that generates salable stool at the demand rate *αe*^*βt*^ is derived in the Laplace domain in Eq. (25) in Additional file 1. Because standard numerical packages had difficulty inverting this equation to derive *r*(*t*), we resorted to a heuristic approach and derived an approximate closed-form expression for *r*(*t*) in Eq. (40) in Additional file 1. This function begins releasing donors 56 days before the beginning of demand to guarantee that salable stool is available to satisfy initial demand on day 0 (Fig. 3a). This release policy generates salable stool at a rate (given by the left side of Eq. (19) in Additional file 1, after solving the system of differential equations in Eqs. (17)–(19) in Additional file 1, with Eq. (40) in Additional file 1 substituted in for *r*(*t*)) that nearly coincides with *αe*^*βt*^ after the first month of demand (Fig. 3b). For time *t*>0 in the nonstationary case, the release rate *r*(*t*) and the demand rate *αe*^*βt*^ are related by *r*(*t*)=*αe*^*βt*^/597. The value of 597 g/donor is smaller than the corresponding value of 958 g per donor in the stationary case, implying that the release rate needs to be more aggressive in the nonstationary case to anticipate increasing demand.

## Discussion

From an operations viewpoint, stool banks are novel in several ways. Compared to blood transfusions and especially organ transplants, stool donors donate to many recipients in an ongoing manner. The explosive demand growth, the need to quarantine processed stools until donors pass a set of stool and serum screens, the uncertainty of a donor’s stool production rate and donation lifetime, and the time delays in receiving stool test results make it challenging to match supply with demand in a cost-effective manner. The goals of the current study are to develop a mathematical framework for describing stool bank operations and to identify policies that improve upon the status quo.

Aside from simply optimizing inter-testing times, we focus on two approaches to improve screening efficiency. First, our exploratory data analysis uncovered considerable heterogeneity of stool production rates across donors, which led us to explore whether this heterogeneity can be exploited by allowing the screening frequency to depend upon an individual donor’s stool production rate. Because many of the test failures are due to two of the 27 agents, which are significantly cheaper and quicker to screen for in isolation, we allow for interim stool testing for just rotavirus and C. difficile. Simultaneously optimizing over both of these options generates a 14.5 % reduction in cost relative to the policy currently used at OpenBiome, which is to perform a regular test (i.e., on all 27 agents) every 60 days. However, a 10.3 % cost reduction can be achieved by using neither of these options but by simply reducing the inter-testing time from 60 to 36 days.

A number of refinements to our model are possible. Openbiome recently instituted a 14-day seroconversion window: when a donor passes a regular test, stools produced in the last 14 days are not released for sale. Hence, only 46 days of stools are released after the first regular test is passed, and 60 days of stools (which includes the last 14 days before the previous test) are released when subsequent tests are passed. We suspect that the inclusion of the 14-day seroconversion window would have a very minor effect on the results presented here.

Several donors have terminated participation at OpenBiome for nonmedical reasons, such as losing interest or moving away. Our model has incorporated these types of failures into the failure rate *γ* and has assumed that they are detected during a stool test, whereas they can actually occur before a scheduled test (in which case a test would be performed before the donor exits the system to determine whether the quarantined stool can be sold). As with the seroconversion window, this modeling omission is likely to have only a minor impact on our results.

Given the novelty of FMT, we have very little data to estimate the failure rates *η* and *γ* and the probability distribution of the stool production rate. As additional data is collected, it should be possible to refine all of the parameter values in Table 1, to determine the nature of the failure process (e.g., whether it has an increasing or decreasing failure rate [11] or whether the exponential distribution suffices) and to observe whether the distribution of stool production rates across donors is smoother than in Fig. 1 in Additional file 1.

Several refinements could alter the optimal screening strategy. Rotavirus is highly seasonal and peaks in winter, and so costs might be reduced with more frequent (interim and regular) screening in winter. Symptom-driven testing, e.g., where an interim test is performed whenever a donor produces two consecutive loose stools (i.e., scoring a 6 or 7 on the Bristol scale [8]), might also reduce costs. Finally, OpenBiome is considering the possibility of bringing screening in house, which would substantially reduce the screening costs and thereby increase the optimal testing frequency (perhaps to the point where it would be optimal to use more than one interim test between regular tests if the latter were restricted to a monthly frequency).

Our model assumes that demand is deterministic. From a modeling point of view, there are two types of demand variability: there may be uncertainty regarding the functional form of the demand (e.g., whether demand increases exponentially or linearly or perhaps as a Bass diffusion model [12], which attempts to capture how FMT is diffusing throughout the health care industry) and its associated parameters, or uncertainty at a more micro level, e.g., where demand is a Poisson process with nonhomogeneous rate *αe*^*βt*^. The operations management literature usually focuses on the latter type of uncertainty [13] because in most cases there are sufficient data available to fit a stochastic demand model. However, in this case—at least at this point in time—the former type of uncertainty probably dominates the latter type of uncertainty, and unforeseen events—such as media coverage or new clinical results—may alter future demand in unexpected ways. In either case, when demand is random, it is possible to either run out of finished goods inventory or to hold excess inventory, and it may be effective to allow the testing interval to be an increasing function of the current finished goods inventory level (i.e., to use expedited testing when there are backorders, in the hope of quickly generating salable stools). A model with random demand could use either a service level approach (e.g., finished goods inventory must be available 95 % of the time) or a cost-based approach, where decisions are made to minimize the expected sum of holding and backorder costs (see [14] for a discussion of these two approaches). Because the difficult-to-quantify loss of goodwill associated with being backordered (e.g., FMTs are delayed or canceled) is likely to be much larger than the out-of-pocket backorder cost (e.g., expedited testing and shipping), the service level approach is probably more practical.

Finally, our model restricts itself to matching the quantity of supply to the quantity of demand. Particularly, as FMT is being considered for chronic diseases such as ulcerative colitis [15], a pressing issue is to match individual donors to individual recipients, perhaps using 16s ribosomal RNA (rRNA) data.

## Conclusion

Key decision variables in stool bank operations are releasing new donors into the system and deciding how frequently to screen donors for infectious agents, which allows their quarantined stools to be released for sale. We find that reducing the inter-testing time from the current value of 60 to 36 days leads to a 10.3 % reduction in costs associated with screening, donating, and processing. Generalizing the screening policy to allow higher-producing donors to be screened more frequently and to add interim stool tests that screen for rotavirus and C. difficile lead to a 14.5 % reduction in cost relative to OpenBiome’s current policy. We also derive a donor release policy that releases new prescreened potential donors into the system so as to produce a supply rate of salable (i.e., post-quarantine) stool that almost exactly satisfies an exponentially-growing demand (Fig. 2b). Our modeling and analysis provide a framework for investigating further improvements of public stool banks.

## Additional file

Additional file 1
**Some model parameters are estimated in Section 1 and the model is analyzed in Section 2.** Figs. 3-4 are discussed in the main text. (PDF 198 kb)
