# Supplementary material for: Optimal screening and donor management in a public stool bank
Source: Microbiome. 2015 Dec 17;3:75. doi: 10.1186/s40168-015-0140-3 (PMC4682266; doi:10.1186/s40168-015-0140-3)
Supplement: Additional file 1 — Some model parameters are estimated in Section 1 and the model is analyzed in Section 2. Figs. 3-4 are discussed in the main text. (PDF 198 kb) [file 40168_2015_140_MOESM1_ESM.pdf]

Additional file 1

Some model parameters are estimated in §1 and the model is analyzed in §2. Figs. 3-4 are discussed in the main text.

1 Parameter Estimation

The PMF of the stool production rate is derived in §1.1, and the test failure rates η and γ are derived in §1.2.

1.1 Stool Production Rate

For each of 30 donors, we compute the donor stool production rate in grams per day, which is the donor's total stool production in grams divided by the total time between the day the donor begins donating and the day the donor exits the system (or until the end of our data collection period if the donor has not exited). We find that the mean production rate is 87.2 grams per day and the coefficient of variation (standard deviation divided by the mean) is 0.74. We use $n = 9$ classes for the intervals $(4.7 - 25.3, 25.3 - 45.9, 45.9 - 66.5, 66.5 - 87.1, 87.1 - 107.7, 107.7 - 128.3, 128.3 - 148.9, 148.9 - 169.7, 169.7 - 190.3)$ grams/day, and set the s_i values to the interval centers $(15, 35.6, 56.2, 76.8, 97.5, 118, 139, 159, 180)$ grams/day to obtain the PMF appearing in Fig. 1.

In addition, we examine how the number of weekly visits by a donor varies over time. For donor $i = 1, \ldots, 30$, let $v_{i\tau}$ be the number of visits by donor i in week τ of the donor's donation period, and let T_i be donor i's lifetime (in weeks) as a donor. The mean (averaged over the number of active donors at each point in time) visit rate in week τ is

$$\frac{\sum_{\{i:T_i \geq \tau\}} v_{i\tau}}{|\{i : T_i \geq \tau\}|}, \tag{1}$$

which is plotted vs. τ in Fig. 2. The mean visit rate is relatively constant through week 49,

and the mean visit rate during weeks 49-54 is unreliable because there are only two donors who have been active for more than 49 weeks.

1.2 Test Failure Rates

The small amount of data precludes us from estimating the exact nature of the time-to-failure distribution. Hence, we assume that the time-to-failure has an exponential distribution, where η is the exponential rate associated with rotavirus and CDI, and γ is the exponential rate associated with the other 25 infectious agents.

The data consist of test results and testing times for 13 active donors. Let D_i^1 be the time between the day donor i begins donating and the day of the donor's first test, and let D_i^2 be the time between donor i's first and second tests. All 13 donors fell into one of the following four categories.

Group 1: Donors who failed their first test due to rotavirus or CDI, which occurs with probability

$$e^{-\gamma D_i^1}(1 - e^{-\eta D_i^1}). \tag{2}$$

Group 2: Donors who failed their first test because of the other 25 agents, which occurs with probability

$$e^{-\eta D_i^1}(1 - e^{-\gamma D_i^1}). \tag{3}$$

Group 3: Donors who passed their first test but failed their second test due to rotavirus or CDI, which occurs with probability

$$e^{-\gamma(D_i^1+D_i^2)}e^{-\eta D_i^1}(1 - e^{-\eta D_i^2}). \tag{4}$$

Group 4: Donors who are still active after passing a single test, which occurs with probability

$$e^{-(\eta+\gamma)D_i^1}. \tag{5}$$

Letting $i \in G_j$ denote that donor i is in group j, we find that the negative log-likelihood associated with our dataset is

$$
\begin{aligned}
L(\eta, \gamma) = & \sum_{i \in G_1} \left[\gamma D_i^1 - \log(1 - e^{-\eta D_i^1}) \right] + \sum_{i \in G_2} \left[\eta D_i^1 - \log(1 - e^{-\gamma D_i^1}) \right] \\
& + \sum_{i \in G_3} \left[\gamma(D_i^1 + D_i^2) + \eta D_i^1 - \log(1 - e^{-\eta D_i^2}) \right] + \sum_{i \in G_4} \left[(\gamma + \eta) D_i^1 \right]. \qquad (6)
\end{aligned}
$$

The function $L(\eta, \gamma)$ in (6) is convex in η and γ, and minimizing this function yields the maximum likelihood values, $\eta = 0.0066$/day and $\gamma = 0.0040$./day

2 Problem Analysis

The problem is formulated in §2.1 in the case of deterministic stationary demand (i.e., $\beta = 0$). In §2.2, we show that the optimal screening strategy in §2.1 also holds for the case of nonstationary demand (i.e., $\beta > 0$).

2.1 Stationary Demand

In the stationary demand case, $\beta = 0$ and the demand rate equals α for all t. Because demand is stationary, we drop the dependence on time t in this subsection. The analysis is performed in two steps: to derive the mean number of donors and the release rate in equilibrium, and then to derive the cost function.

We can think of each donation cycle for a class k donor as having two stages: the first stage consists of the d_k days before the interim test, and the second stage consists of the $D_k - d_k$ days between the interim test and the regular test. Referring to Fig. 1 in the main text, new donors of class k arrive at an average rate of $r f_k p_0 p_s p_b$. After d_k days, class k donors exit the first stage after interim testing, and they fail the interim test (and exit the system) with probability $1 - e^{-\eta d_k}$ and move to the second stage with probability $e^{-\eta d_k}$. Upon entering the second stage, class k donors wait $D_k - d_k$ days and then undergo regular

testing, and they fail the regular test (and exit the system) with probability $1 - e^{\eta d_k - (\eta+\gamma)D_k}$ and return to the first stage with probability $e^{\eta d_k - (\eta+\gamma)D_k}$. Let x_k and y_k be the mean number of class k donors in the first stage and second stage, respectively. Then the above reasoning leads to the system of ordinary differential equations,

$$\dot{x}_k = r f_k p_0 p_s p_b - \frac{1}{d_k} x_k + \frac{e^{\eta d_k - (\eta+\gamma)D_k}}{D_k - d_k} y_k, \tag{7}$$

$$\dot{y}_k = \frac{e^{-\eta d_k}}{d_k} x_k - \frac{1}{D_k - d_k} y_k. \tag{8}$$

Setting the left sides of (7)-(8) to zero and solving yields

$$x_k = \frac{d_k}{1 - e^{-(\eta+\gamma)D_k}} r f_k p_0 p_s p_b, \tag{9}$$

$$y_k = \frac{e^{-\eta d_k}(D_k - d_k)}{1 - e^{-(\eta+\gamma)D_k}} r f_k p_0 p_s p_b. \tag{10}$$

With x_k and y_k in hand, we can compute the release rate r such that the rate at which salable stool is produced equals the demand rate α. Because $s_k D_k$ grams of stool are released from quarantine and made available for sale whenever a class k donor passes a regular test, we have the equation

$$\sum_{k=1}^{n} s_k D_k \frac{e^{\eta d_k - (\eta+\gamma)D_k}}{D_k - d_k} y_k = r p_0 p_s p_b \sum_{k=1}^{n} \frac{s_k D_k f_k}{e^{(\eta+\gamma)D_k} - 1} = \alpha. \tag{11}$$

Solving for r in (11) gives the input rate

$$r = \frac{\alpha}{p_0 p_s p_b \sum_{k=1}^{n} \frac{s_k D_k f_k}{e^{(\eta+\gamma)D_k} - 1}}. \tag{12}$$

Referring again to Fig. 1 in the main text, we can express the total cost per day by

$$C(D_1,\ldots,D_n,d_1,\ldots,d_n) = rc_0 + rp_0c_s + rp_0p_sc_b + \sum_{k=1}^{n}\left((x_k+y_k)c_d + (x_k+y_k)s_kc_p + \frac{x_k}{d_k}c_i + \frac{y_k(c_s + e^{\eta d_k-(\eta+\gamma)D_k}c_b)}{D_k-d_k}\right), \tag{13}$$

where, in the last term in (13), we assume that those failing the regular test will fail the stool test. Substituting (9), (10) and (12) into (13) gives

$$C(D_1,\ldots,D_n,d_1,\ldots,d_n) = \frac{\alpha}{p_0p_sp_b\sum_{k=1}^{n}s_kD_kf_k\frac{e^{-(\eta+\gamma)D_k}}{1-e^{-(\eta+\gamma)D_k}}}\Bigg[c_0 + p_0c_s + p_0p_sc_b + \sum_{k=1}^{n}\frac{f_kp_0p_sp_b}{1-e^{-(\eta+\gamma)D_k}}\left([d_k + e^{-\eta d_k}(D_k-d_k)](c_d+s_kc_p) + c_i + e^{-\eta d_k}c_s + e^{-(\eta+\gamma)D_k}c_b\right)\Bigg]. \tag{14}$$

The optimization problem is given by

$$\min_{D_1,\ldots,D_n,d_1,\ldots,d_n} C(D_1,\ldots,D_n,d_1,\ldots,d_n) \tag{15}$$

$$\text{subject to}\quad D_k \geq d_k \geq 0, \quad 1\leq k\leq n. \tag{16}$$

The cost function in (14) is convex in the domain of interest, and (14)-(16) can be solved via standard convex optimization methods such as projected gradient descent [2]. We round off the solution to the nearest integer to get the results stated in the main text.

2.2 Nonstationary Demand

Turning to the nonstationary case where the demand rate at time t is $\alpha e^{\beta t}$ with $\beta > 0$, we divide the donation cycle into two stages as in §2.1, but we define $x_k(t)$ and $y_k(t)$ differently. Let $x_k(t)$ be the mean number of class k donors who start a new cycle of donation at time

t, where these donors are either new donors who just passed their initial serum screen or continuing donors who just had their previous cycle's stools released form quarantine and offered for sale. Recalling that a class k donor undergoes an interim test d_k days into a cycle and the results from this test incur a delay of τ_i days, we see that the mean number of class k donors who receive their interim test result at day t is $x_k(t-(d_k+\tau_i))$. Let $y_k(t)$ be the mean number of class k donors who receive their regular test results at time t. Because the regular test takes place $D_k - d_k$ days after the interim test and the delay to receive the results of the regular test is τ_s days, the mean number of class k donors who start the second phase of the donation cycle at time t is $y_k(t+(D_k-d_k)+\tau_s)$. By these definitions, on average $e^{\eta d_k-(\eta+\gamma)D_k}y_k(t)$ class k donors pass their regular test and start a new donation cycle at time t, and their produced stool over the last D_k days are released from quarantine and offered for sale. Also, the new class k donors are released into the donation loop at rate $f_k p_0 p_s p_b r(t-\tau_s)$ per day at time t, where τ_s is the time delay incurred by the initial stool test.

Taken together, it follows that the differential equations governing $x_k(t)$ and $y_k(t)$ are

$$\dot{x}_k(t) = y_k(t)e^{\eta d_k-(\eta+\gamma)D_k} + f_k p_0 p_s p_b r(t-\tau_s) - x_k(t), \tag{17}$$

$$\dot{y}_k(t+(D_k-d_k)+\tau_s) = x_k(t-(d_k+\tau_i))e^{-\eta d_k} - y_k(t+(D_k-d_k)+\tau_s), \tag{18}$$

$$\sum_{k=1}^{n} y_k(t)s_k D_k e^{\eta d_k-(\eta+\gamma)D_k} = \alpha e^{\beta t}, \tag{19}$$

where (19) ensures that the nonstationary demand is satisfied.

The above $2n+1$ equations can be solved to derive the $2n+1$ unknown functions $(x_1(t),\ldots,x_n(t),y_1(t),\ldots,y_n(t),r(t))$. We can represent and solve the above equations in the Laplace domain as follows. Let $X_k(z)$, $Y_k(z)$ and $R(z)$ be the Laplace transform of $x_k(t)$,

$y_k(t)$ and $r(t)$, respectively. Then equations (17)-(19) are equivalent to

$$zX_k(z) = Y_k(z)e^{\eta d_k-(\eta+\gamma)D_k} + f_k p_0 p_s p_b R(z)e^{-\tau_s z} - X_k(z), \tag{20}$$

$$zY_k(z)e^{((D_k-d_k)+\tau_s)z} = X_k(z)e^{-\eta d_k}e^{-(d_k+\tau_i)z} - Y_k(z)e^{((D_k-d_k)+\tau_s)z}, \tag{21}$$

$$\sum_{k=1}^{n} Y_k(z)s_k D_k e^{\eta d_k-(\eta+\gamma)D_k} = \frac{\alpha}{z-\beta}. \tag{22}$$

Solving (20)-(21) for X_k and Y_k gives

$$X_k(z) = \frac{e^{(D_k+\tau_s+\tau_i)z}(z+1)}{(z+1)^2 e^{(D_k+\tau_s+\tau_i)z} - e^{-(\eta+\gamma)D_k}} f_k p_0 p_s p_b R(z)e^{-\tau_s z}, \tag{23}$$

$$Y_k(z) = \frac{e^{-\eta d_k}}{(z+1)^2 e^{(D_k+\tau_s+\tau_i)z} - e^{-(\eta+\gamma)D_k}} f_k p_0 p_s p_b R(z)e^{-\tau_s z}, \tag{24}$$

and substituting (24) into (22) yields

$$R(z) = \frac{1}{p_0 p_s p_b} \frac{e^{\tau_s z}}{\sum_{k=1}^{n} s_k D_k f_k \frac{e^{-(\eta+\gamma)D_k}}{(z+1)^2 e^{(D_k+\tau_s+\tau_i)z} - e^{-(\eta+\gamma)D_k}}} \frac{\alpha}{z-\beta}. \tag{25}$$

Now that we have the solution (23)-(25), there are two remaining tasks: attempt to invert equation (25) to obtain the optimal release rate $r(t)$, and derive the cost function and optimal inter-testing times; we begin with the latter task. The total cost rate at time t is

$$\begin{aligned}\hat{c}(t) =& c_0 r(t) + p_0 c_s r(t) + p_0 p_s c_b r(t-\tau_s) + \sum_{k=1}^{n}\Bigg((c_d + s_k c_p)\int_0^{d_k} x_k(t-\tau)d\tau \\ &+ x_k(t-d_k)c_i + (c_d + s_k c_p)\int_0^{D_k-d_k} y_k(t+\tau+\tau_s)\, d\tau + y_k(t)(c_s + c_b e^{\eta d_k-(\eta+\gamma)D_k})\Bigg).\end{aligned} \tag{26}$$

Note that the total cost rate in (26) grows exponentially with time. We re-express our

objective as minimizing the total cost per gram of demanded stool, and let the time horizon go to infinity, i.e.,

$$C(D_1, \ldots, D_n, d_1, \ldots, d_n) = \lim_{T\to\infty} \frac{\int_0^T \hat{c}(t)\ dt}{\int_0^T \alpha e^{\beta t}\ dt}. \tag{27}$$

Let $\hat{C}(z)$ be the Laplace transform of $\hat{c}(t)$, which from (26) is given by

$$\begin{aligned}\hat{C}(z) =& \big(c_0 + p_0 c_s + p_0 p_s c_b e^{-\tau_s z}\big) R(z) + \sum_{k=1}^{n} \Bigg((c_d + s_k c_p) X_k(z) \int_0^{d_k} e^{-\tau z} d\tau \\ &+ X_k(z) e^{-d_k z} c_i + (c_d + s_k c_p) Y_k(z) \int_0^{D_k - d_k} e^{(\tau+\tau_s)z} d\tau + Y_k(z)(c_s + c_b e^{\eta d_k - (\eta+\gamma) D_k}) \Bigg).\end{aligned} \tag{28}$$

Substituting (23)-(25) into (28) gives

$$\hat{C}(z) = G(z) \frac{\alpha}{z - \beta}, \tag{29}$$

where

$$\begin{aligned}G(z) =& \frac{1}{p_0 p_s p_b} \frac{1}{\sum_{k=1}^n s_k D_k f_k \frac{e^{-(\eta+\gamma)D_k}}{(z+1)^2 e^{(D_k+\tau_s+\tau_i)z} - e^{-(\eta+\gamma)D_k}}} \Bigg[\big(c_0 + p_0 c_s + p_0 p_s c_b e^{-\tau_s z}\big) e^{\tau_s z} \\ &+ \sum_{k=1}^{n} \Bigg(\bigg(\Big(e^{-d_k z} c_i + (c_d + s_k c_p) \int_0^{d_k} e^{-\tau z} d\tau \Big)(z+1) e^{(D_k+\tau_s+\tau_i)z} + \Big((c_s + c_b e^{\eta d_k - (\eta+\gamma) D_k}) \\ &+ (c_d + s_k c_p) \int_0^{D_k - d_k} e^{(\tau+\tau_s)z} d\tau \Big) e^{-\eta d_k} \bigg) \frac{p_0 p_s p_b f_k}{(z+1)^2 e^{(D_k+\tau_s+\tau_i)z} - e^{-(\eta+\gamma)D_k}} \Bigg) \Bigg].\end{aligned} \tag{30}$$

Letting $g(t)$ be the inverse Laplace transform of $G(z)$, we re-express (29) in the time domain as

$$\hat{c}(t) = g(t) * (\alpha e^{\beta t}), \tag{31}$$

where $*$ denotes the convolution operation. Substituting (31) for $\hat{c}(t)$ in (27), it follows [3] that

$$C(D_1, \ldots, D_n, d_1, \ldots, d_n) = \int_0^{\infty} g(t) dt. \tag{32}$$

The integral property of the Laplace transform implies that

$$\int_0^\infty g(t)dt = G(0), \tag{33}$$

which together with (30) and (32) give the cost per gram of demanded stool as

$$C(D_1,\dots,D_n,d_1,\dots,d_n) = \frac{1}{p_0p_sp_b\sum_{k=1}^n s_kD_kf_k\frac{e^{-(\eta+\gamma)D_k}}{1-e^{-(\eta+\gamma)D_k}}}\Bigg[c_0+p_0c_s+p_0p_sc_b \\ +\sum_{k=1}^n \frac{f_kp_0p_sp_b}{1-e^{-(\eta+\gamma)D_k}}\left([d_k+e^{-\eta d_k}(D_k-d_k)](c_d+s_kc_p)+c_i+e^{-\eta d_k}c_s+e^{-(\eta+\gamma)D_k}c_b\right)\Bigg]. \tag{34}$$

The cost function in (34) is exactly equal to the cost function (up to the multiplicative constant α) for the stationary case in (14). Therefore, the optimal values of $(D_1,\dots,D_n,d_1,\dots,d_n)$ in the stationary case, given by the solution to the optimization problem (14)-(16), are also optimal for the nonstationary demand case. Furthermore, because we did not use the special structure (i.e., exponential growth) of the nonstationary demand function, the same values of $(D_1,\dots,D_n,d_1,\dots,d_n)$ remain optimal for any arbitrary nonstationary demand function.

Returning to the second task, although numerical methods can be used to compute the optimal release rate $r(t)$ from the optimal release rate $R(z)$ in the Laplace domain, standard numerical packages struggled to invert equation (25). Consequently, in the remainder of this subsection, we describe a heuristic procedure to approximate $r(t)$ from equation (25). The first of two approximations in this heuristic is to replace the n donor classes by a single weighted (with weights $(f_1,\dots,f_n)$) class, denoted by class 0, that has values of s, D and d equal to $s_0=\sum_{k=1}^n f_ks_k$, $D_0=\sum_{k=1}^n f_kD_k$ and $d_0=\sum_{k=1}^n f_kd_k$. Replacing the n classes by class 0 allows us to approximate equation (25) by

$$R_0(z) = \frac{e^{(\eta+\gamma)D_0}}{p_0p_sp_bs_0D_0}\left[(z+1)^2e^{(D_0+\tau_s+\tau_i)z}-e^{-(\eta+\gamma)D_0}\right]\frac{\alpha e^{\tau_s z}}{z-\beta}. \tag{35}$$

Taking the inverse Laplace transform gives the following approximation for $r(t)$:

$$r_0(t) = \frac{\alpha}{p_0 p_s p_b s_0 D_0}\left((\beta+1)^2 e^{(\eta+\gamma)D_0+\beta(D_0+2\tau_s+\tau_i)} H(t+D_0+2\tau_s+\tau_i)e^{\beta t} - e^{\beta\tau_s}e^{\beta t}H(t+\tau_s)\right), \tag{36}$$

where $H(t)$ is the Heaviside step function that requires donors to be released into the system before the start of the demand (i.e., at $t < 0$) to guarantee that there is salable stool to satisfy the initial demand at time 0.

For $t > 0$, equation (36) implies that the demand rate divided by the release rate is given by

$$\frac{\alpha e^{\beta t}}{r_0(t)} = \frac{p_0 p_s p_b s_0 D_0}{(\beta+1)^2 e^{(\eta+\gamma)D_0+\beta(D_0+2\tau_s+\tau_i)} - e^{\beta\tau_s}}, \quad t > 0. \tag{37}$$

Noting that the demand rate divided by the release rate in the stationary case is given by (see equation (12))

$$\frac{\alpha}{r} = p_0 p_s p_b \sum_{k=1}^{n} \frac{s_k D_k f_k}{e^{(\eta+\gamma)D_k} - 1}, \tag{38}$$

we employ the second approximation in our heuristic procedure, which is to re-introduce the n classes into (37) so that equation (37) reduces to equation (38) when $\beta = 0$:

$$\frac{\alpha e^{\beta t}}{r(t)} = p_0 p_s p_b \sum_{k=1}^{n} \frac{s_k D_k f_k}{(\beta+1)^2 e^{(\eta+\gamma)D_k+\beta(D_k+2\tau_s+\tau_i)} - e^{\beta\tau_s}}, \quad t > 0. \tag{39}$$

Rearranging (39) and re-incorporating the Heaviside function gives our approximate release rate,

$$r(t) = \frac{\alpha e^{\beta t}}{p_0 p_s p_b \sum_{k=1}^{n} \frac{s_k D_k f_k}{(\beta+1)^2 e^{(\eta+\gamma)D_k+\beta(D_k+2\tau_s+\tau_i)} H(t+D_k+2\tau_s+\tau_i) - e^{\beta\tau_s}H(t+\tau_s)}}. \tag{40}$$

References

[1] Smith MB, Kassam Z, Burgess J, Perrotta AR, Burns LJ, Mendolia GM, *et al.* The international public stool bank: a scalable model for standardized screening and processing of donor stool for fecal microbiota transplantation. Gastroenterology 2015; 148:S-211,

Abstract Sa1064.

[2] Avriel M. Nonlinear programming: analysis and methods. Englewood Cliffs, NJ: Prentice-Hall; 1976.

[3] Györi I, Horváth L. New limit formulas for the convolution of a function with a measure and their applications. Journal of Inequalities and Applications, 2008; 748929.

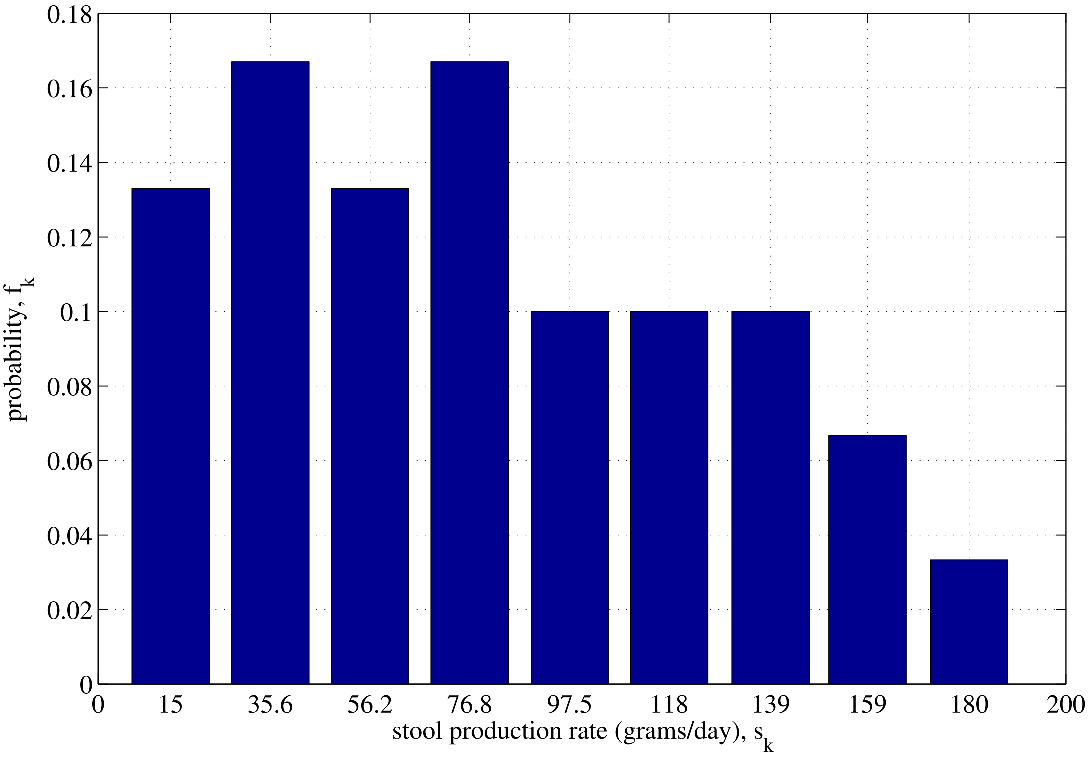

Fig. 1: The PMF f_k of the donor stool production rate (in grams/day). The probabilities that the donor stool production rate equals $(15, 35.6, 56.2, 76.8, 97.5, 118, 139, 159, 180)$ grams/day are $(f_1, f_2, f_3, f_4, f_5, f_6, f_7, f_8, f_9) = (0.1330, 0.1670, 0.1330, 0.1670, 0.1000, 0.1000, 0.1000, 0.0667, 0.0333)$.

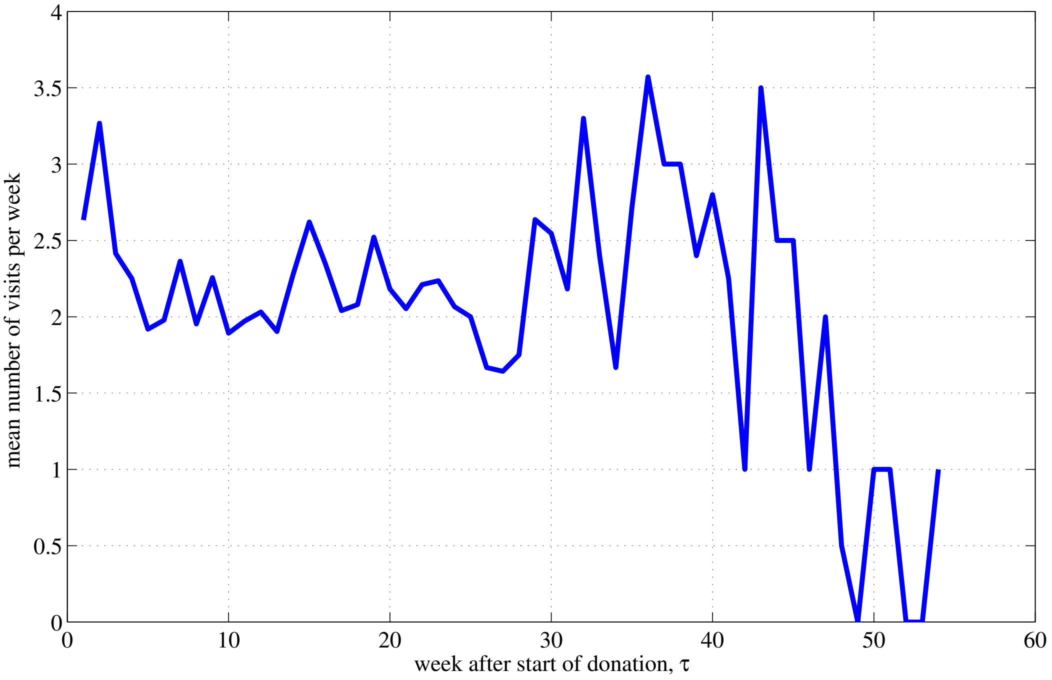

Fig. 2: The mean visit rate (in visits/week) in week τ of the donation period (from equation (1)) vs. τ.

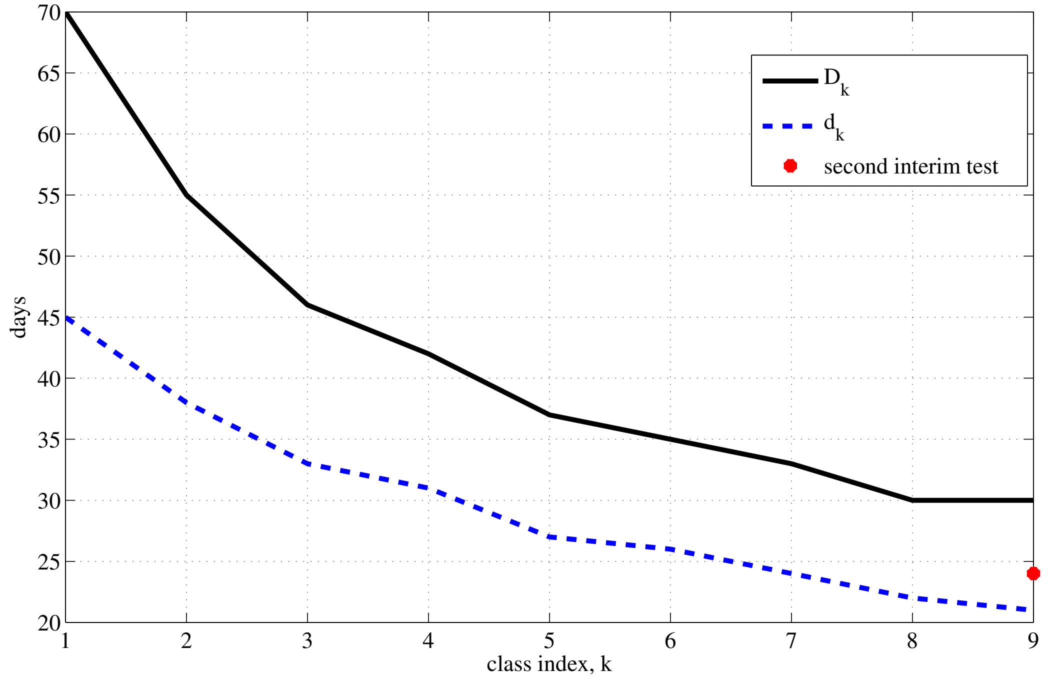

(a)

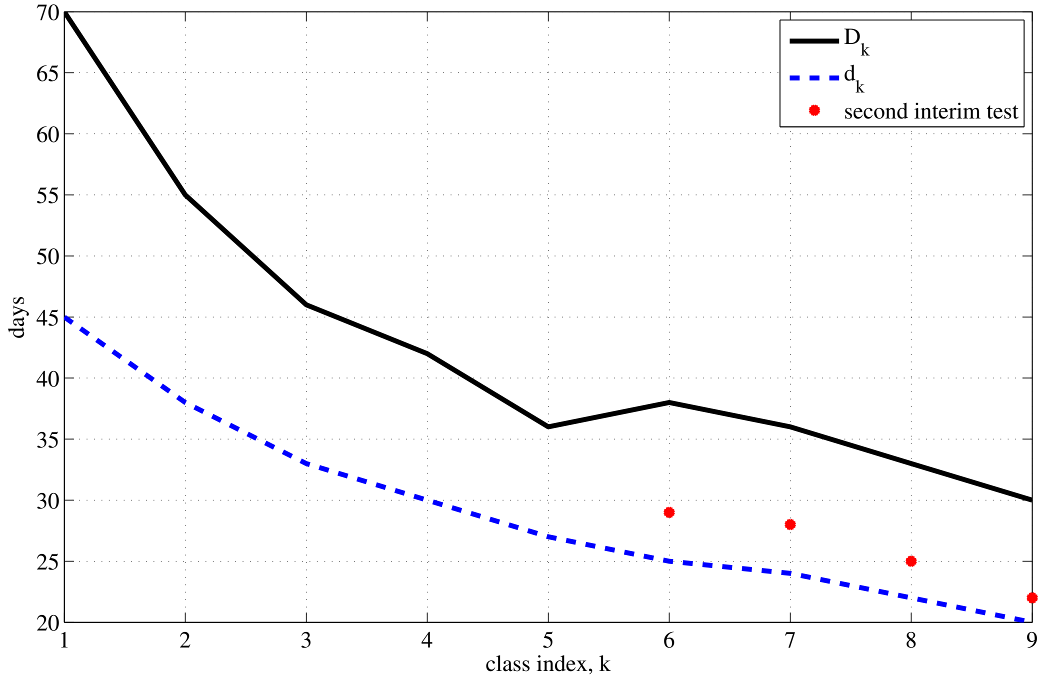

(b)

Fig. 3: The optimal donor-dependent screening variables under the additional constraint $D_k \geq 30$ days. The optimal inter-testing time for regular tests (D_k) and the optimal time between a regular test and an interim test (d_k) as a function of a donor's class, where classes $k = 1, \ldots, 9$ have stool production rates in the intervals $(4.7 - 25.3, 25.3 - 45.9, 45.9 - 66.5, 66.5 - 87.1, 87.1 - 107.7, 107.7 - 128.3, 128.3 - 148.9, 148.9 - 169.7, 169.7 - 190.3)$ grams/day. A second interim test is required **(a)** for class 9, and **(b)** for classes 6, 7, 8 and 9.

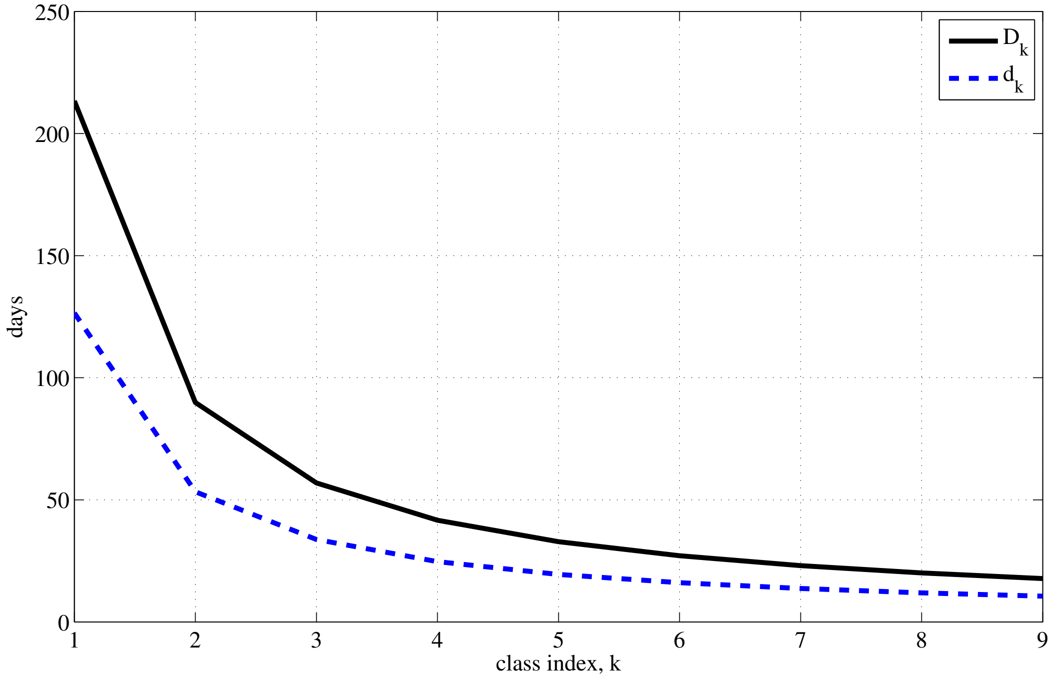

Fig. 4: The optimal donor-dependent screening variables under the two-parameter policy, where $d_k s_k = 1899$ grams and $D_k s_k = 3201$ grams. The optimal inter-testing time for regular tests (D_k) and the optimal time between a regular test and an interim test (d_k) as a function of a donor's class, where classes $k = 1, \ldots, 9$ have stool production rates in the intervals $(4.7 - 25.3, 25.3 - 45.9, 45.9 - 66.5, 66.5 - 87.1, 87.1 - 107.7, 107.7 - 128.3, 128.3 - 148.9, 148.9 - 169.7, 169.7 - 190.3)$ grams/day.
